# Diagnostic Utility of Prominent Mid-Precordial Lead Voltage in a Pediatric Population

**DOI:** 10.1007/s00246-025-03890-w

**Published:** 2025-05-23

**Authors:** Michael White, Mark Gormley, Erick Jimenez, Michael Evans, Bradley Clark

**Affiliations:** 1 Division of Pediatric Cardiology, University of Minnesota, Minneapolis, MN USA; 2 Department of Pediatrics, University of Minnesota, Minneapolis, MN USA; 3 Clinical and Translational Science Institute, University of Minnesota, Minneapolis, MN USA

**Keywords:** Electrocardiogram, Hypertrophy, Voltage, Precordial

## Abstract

ECG criteria for diagnosing chamber enlargement has poor predictive value. Elevated voltages in mid-precordial leads may have clinical utility and we hypothesized that ECGs with a single mid-precordial voltage ≥ 60 mV correlate with congenital heart disease (CHD) in a pediatric cohort. This was a retrospective analysis of pediatric ECGs at the University of Minnesota from 2006 to 2021. Included patients had a single mid-precordial lead (V2-V5) QRS voltage ≥ 60 mV and an echocardiogram within 1 month. ECG parameters including rhythm, atrial enlargement, axis deviation, hypertrophy criteria and echocardiogram findings were evaluated. Of 122 patients (mean 1.6 ± 2.5 years, 62% male), seventeen (14%) (mean 2.5 ± 3.0 years) had normal anatomy and 105 (86%) (mean 1.5 ± 2.4 years) had CHD. Mean maximum QRS voltage was 75.9 ± 13.5 mV with a significantly higher mean maximum voltage in the CHD versus control group (77.0 ± 13.7 vs 69.2 ± 9.7 mV, *p* = 0.012). A receiver operating characteristic curve for maximum QRS voltage had an AUC of 0.691 (95% CI 0.546—0.835). 66.5 mV had the best sensitivity (76%) and specificity (59%) combination and a value of 92.5 mV had a specificity of 100% for CHD. A QRS voltage of 60 mV in mid-precordial leads was associated with CHD in a cohort of pediatric patients. Echocardiograms remain reasonable in these patients though larger cohort studies are needed to develop ideal cutoffs.

## Introduction

The ECG remains a vital yet limited tool in assessing cardiac disease, particularly regarding voltage criteria for chamber hypertrophy and enlargement. These limitations are highlighted in the pediatric population where normal dynamic physiology affects cardiac chambers over time, contributing to differences in normal ECG values based on age. The majority of available studies assessing the predictive utility of ECGs involve patients with normal anatomy and typically target isolated ventricular hypertrophy or lack modern anatomical validation tools. Notably, the Katz-Wachtel phenomenon described by, L.H. Katz, MD and H. Wachtel, MD demonstrates a biphasic QRS complex with greater than 50 mV in the mid-precordial leads or a summation of R and S wave amplitudes greater than 60 mV in leads V3 or V4 were associated with biventricular hypertrophy, and declared pathognomonic for CHD before ultrasound technology was available [1].

Elevated precordial lead voltages are detected frequently in modern ECG screening algorithms, and biphasic QRS complexes with increased total voltage may be found in patients with structurally normal hearts. However, the utilization of these findings has not yet been demonstrated in a cohort of congenital heart disease (CHD) patients. Available research lacks characterizations of specific ECG criteria with confirmatory echocardiography, especially in a range of pediatric age groups. We hypothesize that ECGs with a single mid-precordial lead voltage ≥ 60 mV will correlate with the diagnosis of CHD in a pediatric cohort.

## Methods

**Fig. 1:**
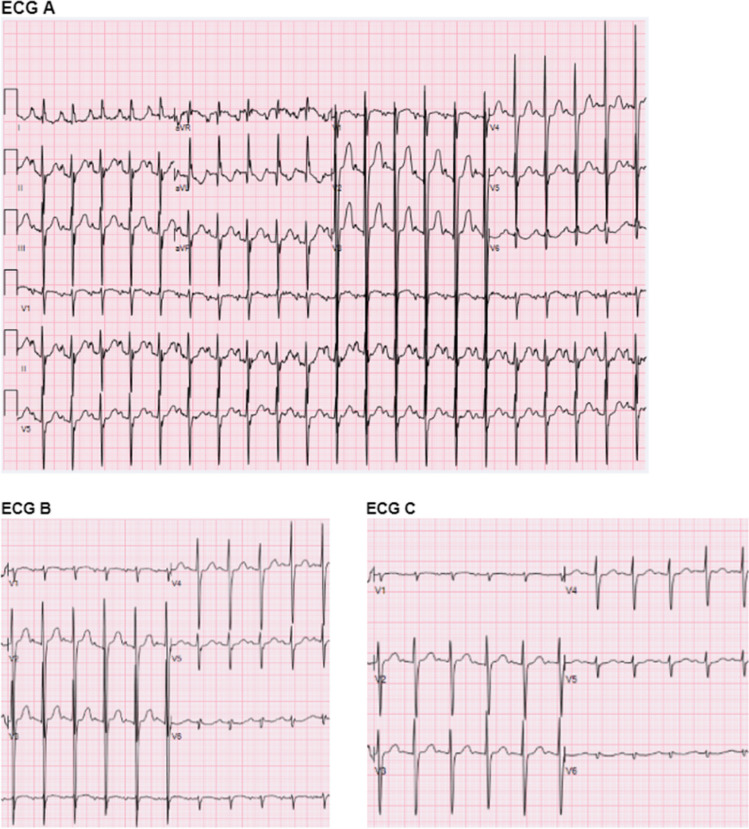
ECG with standard (ECG A), ½ (ECG B) and ¼ (ECG C) gain precordial leads of an 18 month old female with Hypoplastic Left Heart Syndrome variant with apical hypoplasia. Max voltage of 129 mV in V3

This study was approved by the Institutional Review Board at the University of Minnesota. We performed a retrospective analysis of all pediatric (age < 18 years) ECGs performed at the University of Minnesota from 2006 to 2021 via the Muse (GE Healthcare, Chicago, IL) ECG reading software database. Included patients had a single mid-precordial lead (V2-V5) containing a QRS voltage ≥ 60 mV in magnitude, an example demonstrated in Fig. 1, and an echocardiogram performed at our institution within 1 month of the ECG. Of the 352 ECGs evaluated, 122 met inclusion criteria. Each ECG performed was completed at a paper speed of 25 mm/second on the x-axis and voltage in millivolts (mV) on the Y axis. All ECGs and echocardiograms were confirmed by a pediatric cardiologist. Those with a history of orthotopic heart transplant or known cardiomyopathy or metabolic disease were excluded. Patients with a small atrial communication or patent ductus arteriosus were considered normal. Chart review to validate ECG and echocardiogram findings was performed using the electronic medical record. ECG parameters including rhythm, atrial enlargement (AE) criteria, QRS axis deviation (AD) and separate ventricular hypertrophy (VH) criteria and echocardiographic parameters including CHD diagnosis and M-mode measurements, when available, were collected. Definitions of each parameter were defined by age specific criteria and adjudicated by the reading pediatric electrophysiologist [2]. Patient characteristics were summarized using counts and percentages or means and standard deviations, and compared between CHD and normal anatomy using Fisher's exact test or the Wilcoxon rank-sum test. A receiver operating characteristic (ROC) curve was constructed to examine the sensitivity and specificity for CHD of various maximum QRS voltage thresholds. Analyses were conducted using R version 4.2.2.

## Results

**Fig. 2:**
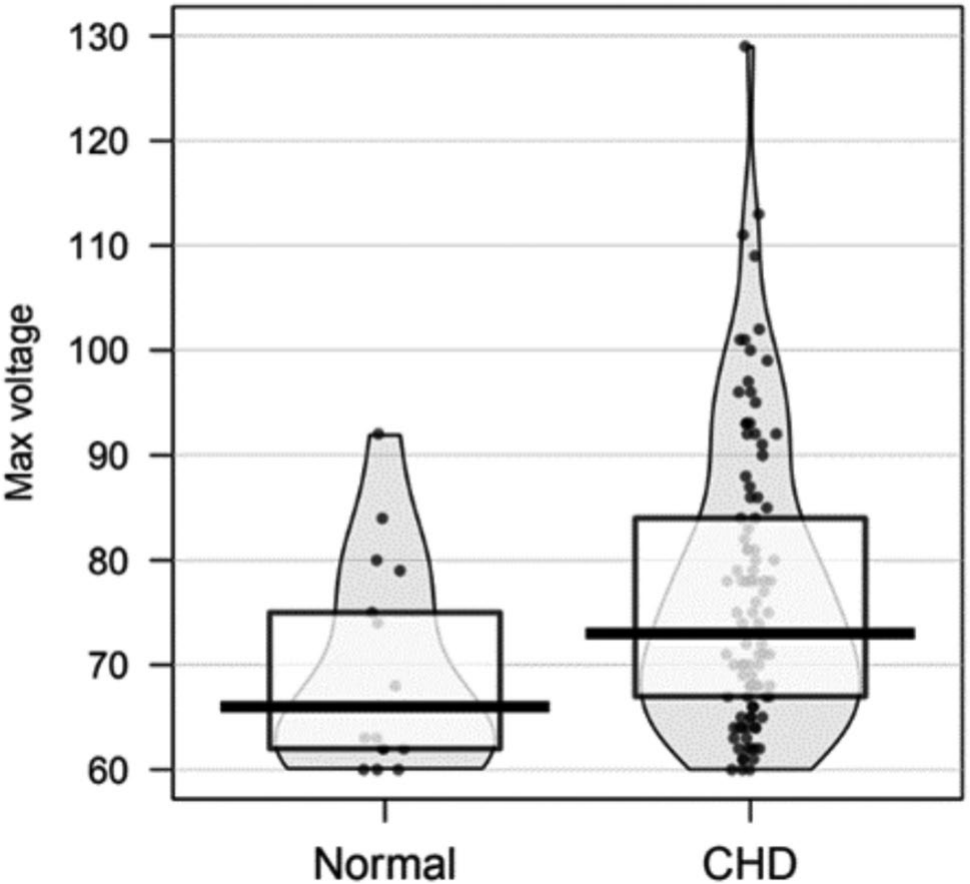
Distributions of max voltages in the normal anatomy and CHD groups, including the medians (black bars), interquartile ranges (boxes), and individual observations (dots). Mean maximum QRS voltage for the cohort was 75.9 ± 13.5 with a significantly higher mean maximum voltage in the CHD versus normal anatomy group (77.0 ± 13.7 vs 69.2 ± 9.7 mV, *p* = 0.012). Although CHD was more present at higher voltages, its presence overlaps with voltages of normal hearts demonstrating the ECGs clinical limitations when used alone and the utilization of a 60 mV cutoff

**Fig. 3:**
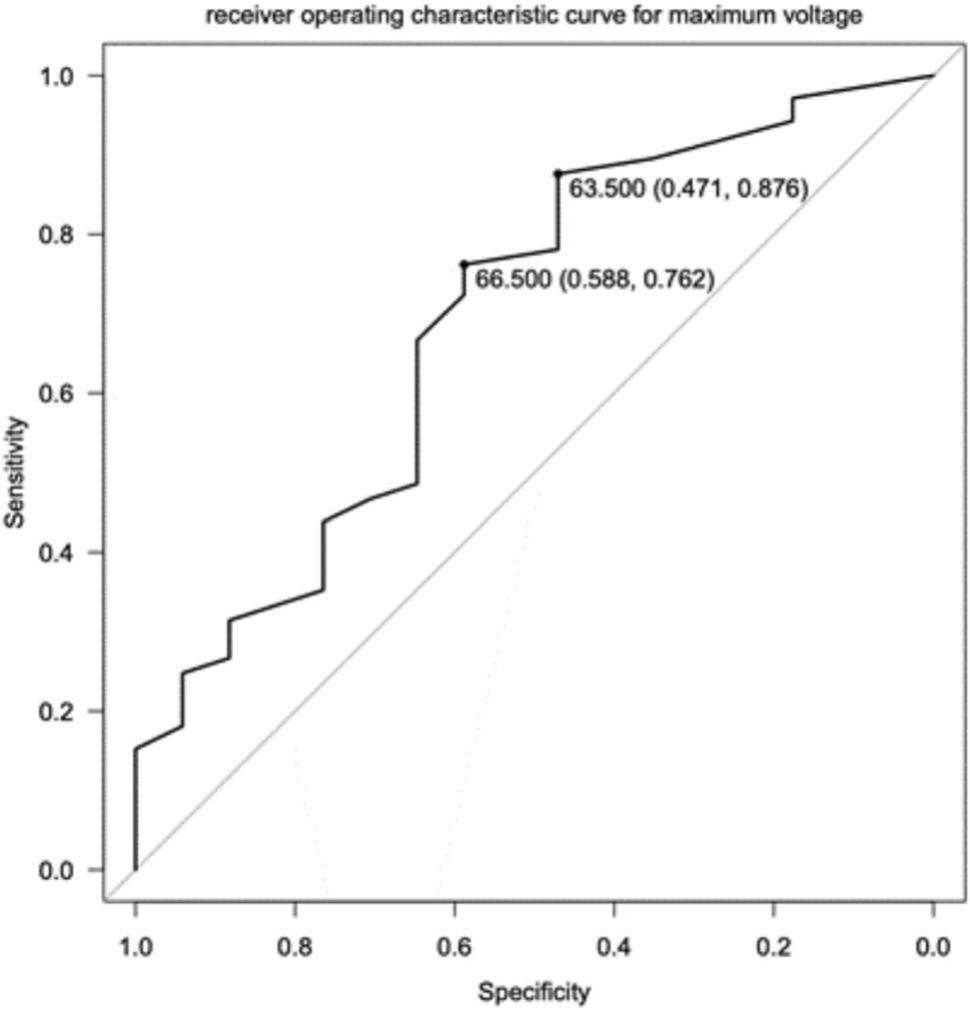
The ROC curve, plotting sensitivity against specificity, helps give thresholds of diagnostic value with the better performing tests being top and left, maximizing sensitivity and specificity. With our cohort the threshold of 66.5 mV maximizes the sum of sensitivity (76%) and specificity (59%). CHD rates were 71% (25/35) and 92% (80/87) in patients below and above the 66.5 mV threshold. When using the conditional inference tree approach, we identified a threshold of 63.5 mV; this had higher sensitivity (88%) but lower specificity (47%), and CHD rates of 62% (13/21) and 91% (92/101) in those below and above this threshold. Despite having less than ideal sensitivity and specificity for clinical practice, these data show CHD is meaningfully present, within a clinical context, in patients with mid-precordial ECG voltages of ≥ 60 mV reiterating the need for confirmatory testing of the possible presence of CHD, when seen on an ECG

Our study included 122 patients (mean 1.6 ± 2.5 years, 62% male). Seventeen (14%) patients (mean 2.5 ± 3.0 years) had normal anatomy and 105 (86%) patients (mean 1.5 ± 2.4 years) had evidence of CHD confirmed via echocardiogram, with ventricular septal defect (*n* = 36, 30%) being the most common lesion. The mean maximum QRS voltage for the cohort was 75.9 ± 13.5 with a significantly higher mean maximum voltage in the CHD versus normal anatomy group (77.0 ± 13.7 vs 69.2 ± 9.7 mV, *p* = 0.012), shown in Fig. 2. A receiver operating characteristic (ROC) curve for maximum QRS voltage was created with an AUC of 0.691 (95% CI 0.546—0.835) demonstrated in Fig. 3. A cutoff value of 66.5 mV had the highest combination of sensitivity (76%) and specificity (59%) in our model and a value of 92.5 mV had a specificity of 100% for diagnosis of CHD. Table 1 demonstrates the sensitivity and specificity of patients that had evidence of AD, AE or VH in combination with mid-precordial lead voltage ≥ 60 mV. Overall the sensitivity of combinations were low, specificities were high including all combinations with AD having specificity of 100%.

**Table 1:** Sensitivity and specificity of various ECG finding and combinations including axis deviation (AD), atrial enlargement (AE) and ventricular hypertrophy (VH) in isolation and combination in addition to the 60 mV cutoff

Criteria	N	Sensitivity	Specificity
AD	39	0.371	0.882
AE	26	0.248	0.941
VH	8	0.076	0.941
AD and AE	13	0.124	1
AD or AE	52	0.495	0.824
AD and VH	5	0.048	1
AD or VH	42	0.4	0.824
AE and VH	2	0.019	1
AE or VH	32	0.305	0.882
AD and AE and VH	2	0.019	1
AD or AE or VH	55	0.524	0.765

When any combination of multiple ECG findings are present, sensitivity tends much lower, yet this gives 100% specificity, and therefore a higher likelihood of CHD identification with an ECG alone

When either criteria were present, sensitivity increased at the expense of sensitivity

## Discussion

Our study demonstrated that ECGs containing a single mid-precordial lead QRS voltage of ≥ 60 mV were strongly associated with a diagnosis of CHD, most commonly an isolated ventricular septal defect. In our cohort, higher voltages had a higher specificity for CHD, and those with CHD on average had higher voltages compared to patients with structurally normal hearts. The cutoff voltage value of 66.5 mV had the highest combination of sensitivity and specificity and all patients with voltages > 92.5 mV were diagnosed with CHD. Notably, sensitivities were slightly higher with AD or AE or VH either in isolation or combination with highest specificities with combinations that included axis deviation. This study highlights the utility of increased mid-precordial lead voltage in the diagnosis of congenital heart disease. Using a voltage cutoff of ≥ 60 mV, most patients had CHD despite the presence of normal anatomy emphasizing the importance of performing a screening echocardiogram for individuals who meet this criterion.

Prior studies characterizing diagnostic utility of ECGs in pediatrics, with various sample sizes, clinical conditions, and study criteria, persistently demonstrated grossly poor sensitivity, specificity, or predictive values. For right ventricular hypertrophy (RVH) assessment (in the context of pulmonary hypertension assessment), Puchalski et al. demonstrated ECGs had a sensitivity of 69%, specificity of 82%, and positive and negative predictive values of 67% and 84% respectively, using RV anterior wall diastolic thickness by [3]. For left ventricular hypertrophy (LVH), Bratincsák et al. showed traditional LVH criteria by ECG, (Q, R or S wave voltage amplitudes exceeding upper limits of normal), poorly correlated with 2D left ventricular mass by echo with ranges of 13–29% for sensitivity, 77–96% for specificity, a positive predictive value (PPV) of 29–50%, and a negative predictive value (NPV) 77% [4]. Similarly for LVH, when using different ECG criteria in combination with left ventricular mass index by echo, Rijnbeek et al. showed low sensitivity of < 25% when elevated left ventricular mass index as reference for LVH, that minimally improved to < 43% when clinical evidence was included, albeit with a 95% specificity [5]. Our data similarly shows poor sensitivity but an increased specificity when mid-precordial voltage is combined with axis deviation, atrial enlargement and additional ventricular hypertrophy. Furthermore, the utilization of the ROC curve and 92.5 mV cutoff value with 100% specificity, may be a useful strategy for evaluating pediatric ECGs with a high rate of CHD diagnosis.

Without concerning physical exam findings, providers at times rely on ECG findings to decide the utility of a screening echocardiogram, though the sensitivity of those approaches vary wildly.

Alexander et al. recently evaluated a large cohort of > 2,000 healthy children with screening digital ECGs and found that there was weak correlation between multiple ECG measures and echocardiographic measures of left ventricular size [6]. The use of axis deviation alone, specifically left axis deviation, had a low diagnostic yield including 15% in one pediatric cohort and 1.4% in another study when it included a normal physical exam [7, 8]. Further, Ravi et al. performed financial analyses and found the cost to find CHD in patients with left axis deviation and a normal physical exam was more than $8,000 [9]. Dasgupta et al. evaluated the appropriate use criteria (AUC) for outpatient pediatric echocardiography [9]. Which, includes a non-specific definition of abnormal ECG and found only a 6.5% diagnostic yield of congenital heart disease which was mostly comprised patients with secundum atrial septal defects [10]. Though the finding of prominent mid-precordial voltage is rarer than the findings of axis deviation or more standard hypertrophy definitions, the diagnostic yield is clearly significantly higher and should be included in standard pediatric ECG evaluation.

Notable limitations of the study include the retrospective nature of the study which can affect data collection conditions and methods. Certain physiologic conditions such as edema, respiratory variation or support, sweat and body habitus can affect ECG measurements, particularly ECG voltages. Therefore it is possible that there were patients with lower voltages than our cutoff which would have impacted the diagnostic utility of the 60 mV value. Our numbers are relatively low though despite appropriate statistical analysis there may be somewhat limited clinical utility. ECGs with voltages less than 60 mV were not retrospectively analyzed nor included due to the volume of patients vastly exceeding and being unrelated to our target population. Additional ECG criteria outside of AD, AE, and VH, such as T wave abnormalities were not included to further assess the utility of a 60 mV mid-precordial lead QRS voltage, however, this warrants further investigation as a potential additional parameter. Echocardiograms can be performed with different machines with potential for poor images limiting measurements though the impact is limited since our study focused on the diagnosis of congenital heart disease. Each echocardiogram and ECG was confirmed by a pediatric cardiologist which should limit the impact of poor studies or those done with different machines or software.

## Conclusion

Our study demonstrated a minimum QRS voltage of 60 mV in a mid-precordial lead is frequently associated with CHD in a pediatric cohort though can be present amongst structurally normal hearts. Although the sensitivity remains low in this cohort, specificity is high and addition of axis deviation, atrial enlargement and separate ventricular hypertrophy criteria increases diagnostic certainty. Despite poor sensitivity, a screening echocardiogram remains reasonable in this population though further study is needed in order to determine whether there is an ideal cutoff value for maximum QRS voltage that reliably predicts the presence of CHD.

## Data Availability

No datasets were generated or analysed during the current study.
